# Enabling point-of-care testing and personalized medicine for schizophrenia

**DOI:** 10.1038/s41537-016-0005-1

**Published:** 2017-01-05

**Authors:** Paul C. Guest, Daniel Martins-de-Souza

**Affiliations:** Laboratory of Neuroproteomics, Department of Biochemistry and Tissue Biology, Institute of Biology, University of Campinas (UNICAMP), Rua Monteiro Lobato 255, Cidade Universitária Zeferino Vaz, Campinas, 13083-862 Brazil

Schizophrenia affects around 1% of the people in the world at some point in their lifetime. This disease can impair quality of life, wellbeing and productivity, along with significant secondary effects on society, the work force, the healthcare services and the general economy.^1^ The problem is compounded by the fact that current medications for schizophrenia are only effective around half of the time and they often have serious side effects.^2,3^ Potentially the major hindrance to improvements in this area is that the diagnosis of schizophrenia has remained essentially unchanged over the decades and is still based on manifestation of subjective symptoms. Thus, diagnoses can differ significantly as these rely on training and adherence of clinicians to loose criteria outlined in the current diagnostic manuals as the DSM-5 and ICD-10. The lack of knowledge about the underlying pathophysiology of schizophrenia has also contributed to the high attrition rate in the development of new drugs and the consequential near abandonment of further efforts in this area by the major drug companies.^4^

Despite the urgent needs for improved diagnoses and treatments, no clinically validated molecular biomarker tests have been developed for schizophrenia or any other psychiatric disorder. There is now an increasing interest for new technologies and biomarker-based approaches, which are fast, cost-effective and simple to use for clinical scientists and technicians. The use of accurate in vitro diagnostic devices that can accurately classify patients according to the type or sub-type of a psychiatric disorder will help to reduce the time spent in the untreated illness phase and improve compliance by placing the right patients on the right therapeutics as early as possible in the disease course. Several means of accomplishing this challenging task have emerged over the last decade. These include portable mass spectrometry devices,^5^ lab-on-a-chip (LOC)^6^ and smartphone applications (apps),^7^ which can return results in less than 15 min from a single drop of blood. Thus far, none of these instruments have been used for measurement of biomarkers in schizophrenia although this is likely to change in the near future considering their success in other medical areas and the critical need for such inexpensive, point-of-care devices in psychiatric medicine.

With this goal in mind, there have now been developments of multiplex biomarker tests on smartphone-based devices, using printed opto-mechanical interfaces for high throughput imaging of test samples.^8^ The data can be transmitted to a database for analysis and the results returned to the user in less than one minute. This mobile platform has already proven successful in clinical settings through rapid analysis of multiplex assays for several infective viruses. In each case, these use the smartphone camera optics function for accumulation and transmission of the readouts. It is likely that other LOC and smartphone-based system will be developed for other disease areas such as neurological and psychiatric disorders like schizophrenia. Such an approach will allow persons to be treated based on their individual biomarker profiles rather than as one of many with a particular disease using a standard blockbuster drug. In addition, the use of multiplex biomarker tests on LOC devices and other handheld instruments which are capable of distinguishing disease subtypes may be useful for rapid identification of patients who are most likely to respond to specific medications. In schizophrenia, this could result in more effective treatment of patients with fewer side effects and, therefore, better overall treatment outcomes.
